# Involvement of Ethylene in the Latex Metabolism and Tapping Panel Dryness of *Hevea brasiliensis*

**DOI:** 10.3390/ijms160817885

**Published:** 2015-08-04

**Authors:** Riza-Arief Putranto, Eva Herlinawati, Maryannick Rio, Julie Leclercq, Piyanuch Piyatrakul, Eric Gohet, Christine Sanier, Fetrina Oktavia, Julien Pirrello, Kuswanhadi, Pascal Montoro

**Affiliations:** 1 Centre International de Recherche Agronomique pour le Développement, Unité Mixte de Recherche Amélioration Génétique & Adaptation des Plantes Méditerranéennes et Tropicales, F-34398 Montpellier, France; E-Mails: rizaputranto@iribb.org (R.-A.P.); maryannick.rio@cirad.fr (M.R.); julie.leclercq@cirad.fr (J.L.); nuch1505@yahoo.com (P.P.); christine.sanier@cirad.fr (C.S.); julien_pirrello@yahoo.fr (J.P.); 2 Indonesian Research Institute for Biotechnology and Bioindustry, Bogor 16128, Indonesia; 3 Indonesia Rubber Research Institute, Sembawa Research Centre, Palembang 30001, Indonesia; E-Mails: eva_herlinawati@yahoo.com (E.H.); fetrina_oktavia@yahoo.com (F.O.); 4 Rubber Research Institute of Thailand, Chatuchak, Bangkok 10900, Thailand; 5 Centre International de Recherche Agronomique pour le Développement, Unité de Recherche Performance des Systèmes de Culture des Plantes Pérennes, F-34398 Montpellier, France; E-Mail: eric.gohet@cirad.fr; 6 Indonesia Rubber Research Institute, Bogor 16151, Indonesia

**Keywords:** abiotic stress, antioxidant, ethephon, ethylene response factor, oxidative stress, rubber, transcription factor

## Abstract

Ethephon, an ethylene releaser, is used to stimulate latex production in *Hevea brasiliensis*. Ethylene induces many functions in latex cells including the production of reactive oxygen species (ROS). The accumulation of ROS is responsible for the coagulation of rubber particles in latex cells, resulting in the partial or complete stoppage of latex flow. This study set out to assess biochemical and histological changes as well as changes in gene expression in latex and phloem tissues from trees grown under various harvesting systems. The Tapping Panel Dryness (TPD) susceptibility of *Hevea* clones was found to be related to some biochemical parameters, such as low sucrose and high inorganic phosphorus contents. A high tapping frequency and ethephon stimulation induced early TPD occurrence in a high latex metabolism clone and late occurrence in a low latex metabolism clone. TPD-affected trees had smaller number of laticifer vessels compared to healthy trees, suggesting a modification of cambial activity. The differential transcript abundance was observed for twenty-seven candidate genes related to TPD occurrence in latex and phloem tissues for ROS-scavenging, ethylene biosynthesis and signalling genes. The predicted function for some Ethylene Response Factor genes suggested that these candidate genes should play an important role in regulating susceptibility to TPD.

## 1. Introduction

The gaseous plant hormone ethylene regulates numerous developmental and physiological processes including fruit ripening, flower induction, senescence and responses to biotic and abiotic stress. The ethylene biosynthesis and signalling pathways have been well established in plant model species. Ethylene has a wide variety of applications in agriculture and horticulture. The effect of ethephon, an ethylene releaser, was tested as early as the 1970s on natural rubber production [1]. The polymer *cis*-1,4-polyisoprene, known as natural rubber, is synthesized in the rubber particles of laticifers, which are articulated and anastomosed latex cells [2,3]. Latex, a cytoplasmic component of laticifers, is a colloidal suspension that contains 30% to 50% dry matter, of which 90% is rubber [4]. Latex is expelled after bark tapping (cutting of soft bark). For certain rubber clones with a low latex metabolism, application of ethephon to the bark stimulates latex flow and latex regeneration between two tappings [5]. The mechanism of ethylene stimulation on latex yield has been shown to be involved in regulating latex metabolisms in many ways [6]. Bark wounding by tapping causes endogenous production of ethylene, which activates the isoprenoic metabolism [6].

Ethylene is also associated with negative effects, such as the activation of nicotinamide adenine dinucleotide phosphate (NADPH) oxidase located at the surface of lutoids (vacuo-lysosomal particles), which generates reactive oxygen species (ROS) [7]. Excessive environmental and harvesting stress induces an overproduction of ROS that cannot be overcome by ROS-scavenging systems. High production of anion superoxide generated at the surface of lutoids leads to lipid peroxidation of the membrane [7]. ROS damages lutoid membranes, thereby jeopardizing their integrity and resulting in the cessation of latex production [7,8]. This lutoid decompartmentalization leads to the release of various types of proteins involved in the agglutination of rubber particles. Hevein, a lectin-like protein, is likely to play a role in the latex coagulation mechanism by bridging between rubber particles [9].

**Figure 1:**
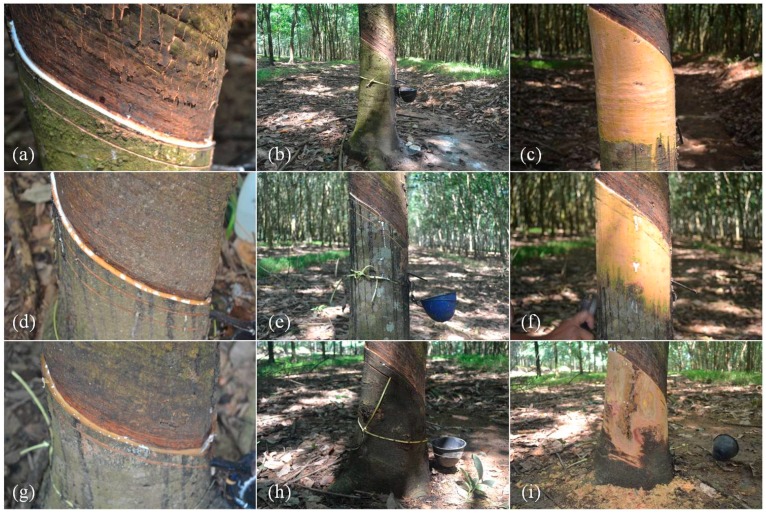
Illustration of Tapping Panel Dryness (TPD) symptoms in *Hevea brasiliensis* plantation. (**a**) Normal latex flow on tapping panel; (**b**) Healthy bark; (**c**) Healthy bark after scrapping; (**d**) Partial cessation of latex flow related to reactive oxygen species-Tapping Panel Dryness (ROS-TPD); (**e**) ROS-TPD bark; (**f**) ROS-TPD bark after scrapping; (**g**) Total cessation of latex flow related to brown bast-TPD (BB-TPD); (**h**) BB-TPD bark; (**i**) BB-TPD bark after scrapping.

The Tapping Panel Dryness (TPD) syndrome is characterized by the partial or, ultimately, total stoppage of latex flow upon tapping, due to *in situ* coagulation of rubber particles. Numerous studies have shown that TPD appears to be a physiological disorder resulting from excessive recurrent tapping and overstimulation by ethylene [6,7,8,10,11]. The term TPD is related to two types of physiological symptoms. Firstly, a temporary halt in latex flow is reversible after a resting period for the trees (Figure 1d–f) [8]. This form of TPD is related to an overproduction of reactive oxygen species (ROS) in laticifers, called ROS-TPD in this manuscript. In aggravated conditions, an irreversible-type of TPD occurs that is called brown bast TPD (BB-TPD) (Figure 1g–i). The latter involves histological deformation of bark due to thylosoid formation, lignified gum, and abnormal division of parenchyma cells, and may be related to a cyanogenesis process [12,13,14,15].

TPD susceptibility depends on the planting material (genetic susceptibility and rootstock-scion interaction) and the environment (harvesting system, abiotic and biotic stress, soil compaction, *etc.*) [16,17]. So the choice of clones and harvesting systems (management of tapping panel, tapping frequency, ethephon stimulation) must be carefully adapted to the eco-climatic conditions and intrinsic latex metabolism of the rubber clone. TPD-affected trees can be cured by bark scraping and the application of chemicals. Tapping can be reconsidered after a resting period up to bark regeneration. However, this process is costly and a year of latex production can be lost. In order to prevent high TPD occurrence, latex diagnosis is used to monitor the physiological status of rubber trees and adjust harvesting systems [18].

Latex diagnosis consists in measuring four main parameters: sucrose, inorganic phosphorus (Pi) and thiol contents, and the total solid content (TSC) of latex [18,19]. Sucrose is the source of carbon for the biosynthesis of *cis*-1,4-polyisoprene. A high sucrose content must be maintained to regenerate latex after latex flow and avoid any cell dysfunction that can lead to TPD [20]. The inorganic phosphorus content reflects the turn-over of ATP and consequently metabolic activity. The thiol content is a parameter to check for the detoxification capacity of laticifers. Lastly, TSC is a way of estimating the dry rubber content, hence the actual production of rubber trees. This latex diagnosis leads to the definition of a clonal typology used to classify rubber clones and to predict the most suitable harvesting system for each one [18,21]. For instance, high sucrose and low Pi contents indicate that a rubber clone has a low latex metabolism and a high latex production potential [21]. This kind of clone requires ethephon application to stimulate its latex metabolism. These low metabolism clones (e.g., Prang Besar (PB) 217 and Proefstation voor Rubber (PR) 107) are more TPD-tolerant [8,22]. By contrast, clones with low sucrose and high Pi contents (e.g., PB 260 and PB 235) are considered to have an active metabolism and are more susceptible to TPD [21,23].

Many ethylene-responsive genes have been related to these physiological changes. More recently, transcriptome analyses led to the identification of candidate genes and microRNAs related to TPD occurrence [24,25,26,27,28]. Ethylene and ROS-scavenging-related genes were for long suggested to play an essential role in TPD occurrence. These genes were recently identified and characterized in response to various types of abiotic and harvesting stress [29,30,31,32] but not yet for TPD occurrence.

Our objective was to provide a basis for understanding the molecular regulation of ethylene biosynthesis and signalling pathways, as well as the ROS-scavenging system during the TPD process. We used morphological, histological and biochemical analyses to classify three contrasting rubber tree clones according to their latex metabolism and TPD susceptibility. Latex production and the dry cut length were measured over a period of 3 years, revealing the higher TPD susceptibility of clone PB 260 compared to other tested clones. Data from latex diagnoses showed a gradient of metabolic typology between the three clones studied (PB 260, Rubber Research Institute of Malaysia (RRIM) 600, and Sungei Putih (SP) 217). A decrease in some biochemical components revealed lower latex metabolism activity in TPD-affected trees than in healthy trees. At the same time, we characterized the regulation of genes related to ethylene biosynthesis and signalling pathways, and ROS-scavenging systems. This study provides insight into the transcriptional regulation of ethylene-related and ROS-scavenging genes in rubber-producing tissues. The comparison with model species led us to propose some candidate genes that could be used to identify molecular markers associated with TPD tolerance for breeding programmes.

## 2. Results

### 2.1. Metabolic Typology of Three Rubber Clones with Contrasting Latex Metabolism

**Figure 2:**
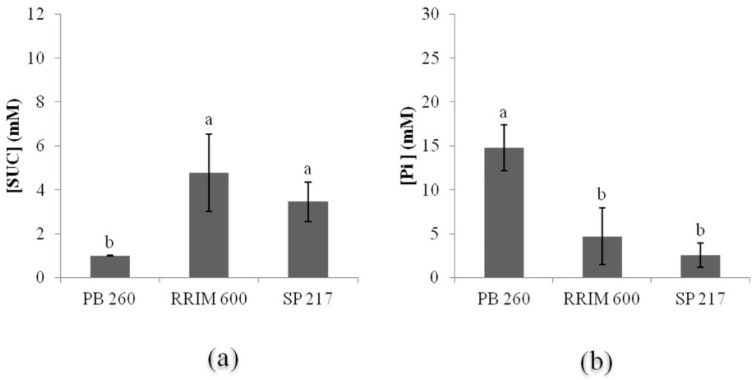
Metabolic typology for three rubber clones (PB 260, RRIM 600, and SP 217) with contrasted latex metabolism based on sucrose (SUC) and inorganic phosphorus (Pi) content in latex. The ANOVA test and the Newman–Keuls test were used in the statistical analyses (*p* < 0.05). (**a**) Sucrose content; (**b**) Inorganic phosphorus.

The metabolic typology of three rubber clones with a contrasting latex metabolism (PB 260, RRIM 600, and SP 217) was checked by a latex diagnosis analysis. Latex samples were analysed from trees tapped every two days and without ethephon stimulation after one year of tapping (Figure 2a,b). Rubber clone PB 260 had a lower sucrose content (1.00 mM) and a higher Pi content (14.78 mM) than clones RRIM 600 (4.77 and 4.70 for sucrose and Pi, respectively) and SP 217 (3.46 and 2.54 for sucrose and Pi, respectively). Clone PB 260 clearly showed low carbon source availability and high metabolism energy revealing its susceptibility to ethephon stimulation and consequently to TPD. The other clones clearly had a lower latex metabolism that should withstand ethephon stimulation.

### 2.2. Evolution of Latex Diagnosis Parameters in Response to Various Harvesting Systems for Three Rubber Clones with Contrasting Latex Metabolism

**Figure 3:**
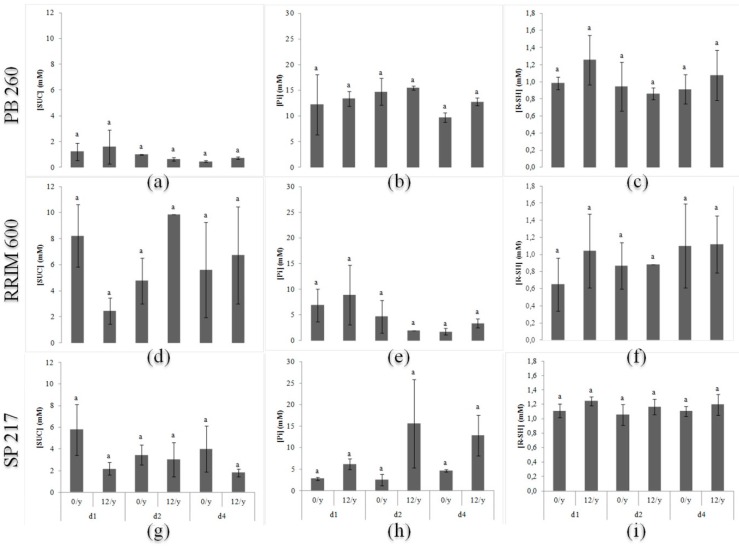
Sucrose (SUC; **a**, **d**, **g**), inorganic phosphorus (Pi; **b**, **e**, **h**) and thiol (R-SH; **c**, **f**, **i**) contents for three rubber clones (PB 260, RRIM 600, and SP 217) with contrasting latex metabolism 12 months after first tapping. The ANOVA test and the Newman–Keuls test were used in the statistical analyses (*p* < 0.05).

The sucrose content peaked at 1.6 mM in latex from clone PB 260 under the d1 ET 12/y treatment, whereas it could reach 5.79 and 9.84 mM for clones SP 217 and RRIM 600 under d1 ET 0/y and d2 ET 12/y, respectively (Figure 3a,d,g). The tapping frequency and ethephon application did not show any significant effect on sucrose content. The inorganic phosphorus content ranged from 9.76 to 14.78 mM in latex from clone PB 260 (Figure 3b). By contrast, latex (Pi) was low for clones RRIM 600 and SP 217, except for some specific treatments: 6.91–8.90 mM for clone RRIM 600 for the d1 tapping frequency only, and 12.92–15.64 mM for clone SP 217 under tapping frequencies d2 and d4 with ET 12/y stimulation (Figure 3e,h). A high tapping frequency (d1) and ethephon application activated the latex metabolism of clones RRIM 600 and SP 217, which had a low intrinsic latex metabolism observed under d2 without stimulation. The thiol content ranged from 0.65 to 1.26 mM whatever the clone, and no treatment effect could be significantly detected (Figure 3c,f,i).

### 2.3. Occurrence of Tapping Panel Dryness in Three Rubber Clones with Contrasting Latex Metabolism

**Figure 4:**
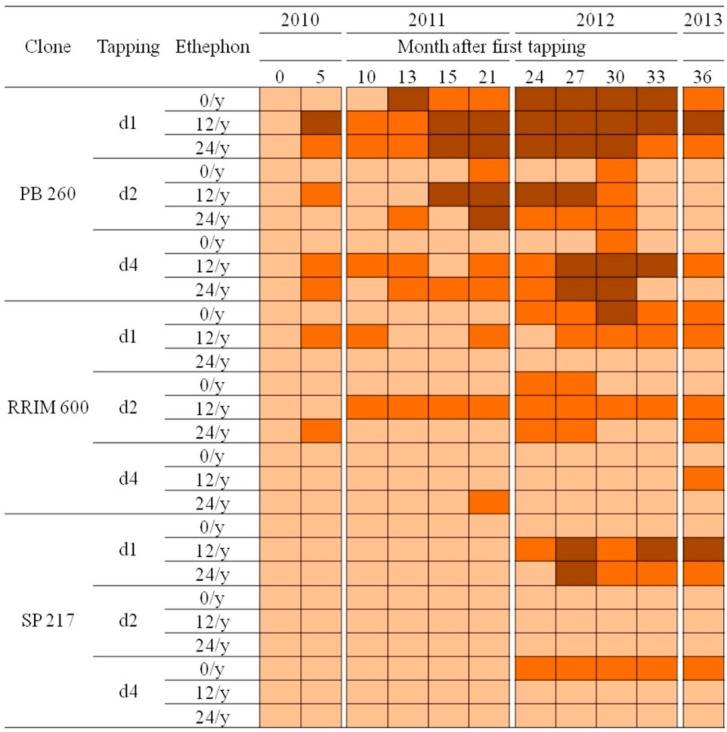
Dry cut length (DCL) measurements for three rubber clones (PB 260, RRIM 600, and SP 217) with contrasting latex metabolism during 36 months of observation. The data was presented by light brown, medium brown, and dark brown colours representing 0%–33%, 34%–66%, and 67%–100% of DCL, respectively.

In order to predict the occurrence of TPD for each studied clone, the dry cut length was measured over a period of three years (Figure 1a,d,g and Figure 4). Clone PB 260 revealed an early dry cut symptom (34%–100%) 5 months after the first tapping for several treatments (d1 ET 12/y, d1 ET 24/y, d2 ET 12/y, d4 ET 12/y, d4 ET 24/y). The severity of this symptom was stronger for treatments with a high tapping frequency (d1). Reversibility of the TPD symptom was observed for treatments with a d2 and d4 tapping frequency. Clone RRIM 600 revealed differential dry cut occurrence: 34%–66% at 5 months after the first tapping for d1 ET 12/y and d2 ET 24/y; 34%–66% at 10 months for treatment d1 ET 12/y and d2 ET 12/y, which was maintained up to 36 months. By contrast, clone SP 217 revealed late dry cut occurrence 24 months after the first tapping for treatments d1 ET 12/y and d4 ET 0/y and 27 months after for treatment d1 ET 24/y.

The interaction between clones, tapping and ethephon frequencies was analysed for the dry cut length (Table 1). A clone effect was significantly observed from 4 months to 2 years after tapping. Meanwhile, no combination effect was significantly observed. The effect of tapping or ethephon was significant in the third year. A detailed analysis revealed that clone PB 260 was significantly more susceptible to TPD than the other clones in the first 2 years after tapping (Table S1). No difference was significantly observed between clones RRIM 600 and SP 217. The effects of tapping and ethephon were significant only after three years of tapping and were consequently greater than the clone effect (Tables S2 and S3). At the late stages of TPD (more than 3 years after the first tapping), the differences between clones were no longer significant.

**Table 1:** Interaction between the effect of genotype, tapping, ethephon and the combination of factors during 4 years. The ANOVA test and the Bonferroni test were used in the statistical analyses (*p* < 0.05). Significant data was marked with an asterisk.

Source	Degree of Freedom	Year			
		2010	2011	2012	2013
		F	F	F	F
Clone	2	11.244 *	10.795 *	3.498 *	0.595
Tapping	2	0.87	0.798	1.899	7.535 *
Ethephon	2	17.25	2.633	0.927	6.579 *
Clone * Tapping	4	0.42	2.253	2.219	1.203
Clone * Ethephon	4	0.661	0.893	1.049	0.301
Tapping * Ethephon	4	0.665	1.254	0.216	0.518
Clone * Tapping * Ethephon	8	0.53	1.046	0.543	1.859

### 2.4. Production of Dry Rubber during the Occurrence of TPD

**Figure 5:**
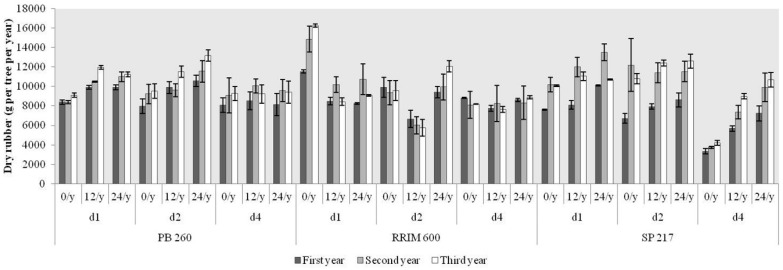
Rubber production for three rubber clones with contrasting latex metabolism during 2 years of observation after first tapping. Values are total mean of 12 months of production. Standard deviation (black bar).

In order to estimate yield production, dry rubber was calculated for each year during the observation (Figure 5). The highest rubber production was 14,875 gram of dry rubber per tree per year for clone RRIM 600 with treatment d1 ET 0/y as opposed to the lowest one with 3334 for clone SP 217 with treatment d4 ET 0/y (Table S4). No clone effect was observed during three years of rubber production (Table S5). The effect of tapping was observed for treatment d1 and d4 at second and third year of production (Table S6). Finally, the different frequency of ethephon stimulation did not give significant difference in rubber production in the observation during this study (Table S7).

### 2.5. Evolution of Latex Diagnosis Parameters in the Latex of Healthy and TPD-Affected Trees

**Figure 6:**
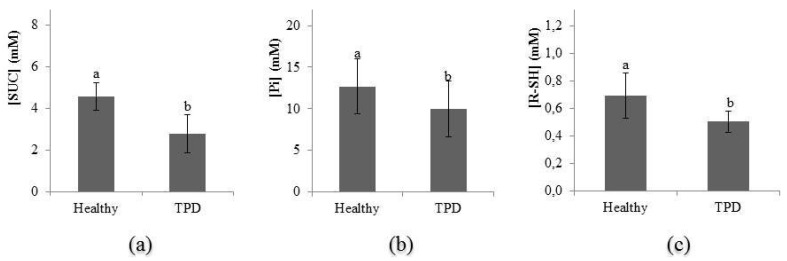
Sucrose (SUC; **a**), inorganic phosphorus (Pi; **b**) and thiol (R-SH; **c**) contents in latex of healthy and TPD-affected plants for clone PB 260. The Fisher’s *F*-test was used in the statistical analyses (*p* < 0.05).

In a previous analysis, susceptibility to TPD was shown in three contrasting clones in which clone PB 260 was the most susceptible compared with the other two clones. Regardless of tapping frequency and ethephon stimulation, a latex diagnosis was carried out three years after the first tapping on healthy and TPD-affected trees from this clone (Figure 6a–c). The sucrose (2.77 instead of 4.56 mM), inorganic phosphorus (9.98 instead of 12.70 mM), and thiol (0.51 instead of 0.70 mM) contents were significantly lower in TPD-affected trees compared to healthy trees.

### 2.6. Anatomical Study of Rubber Tree Soft Bark

**Figure 7:**
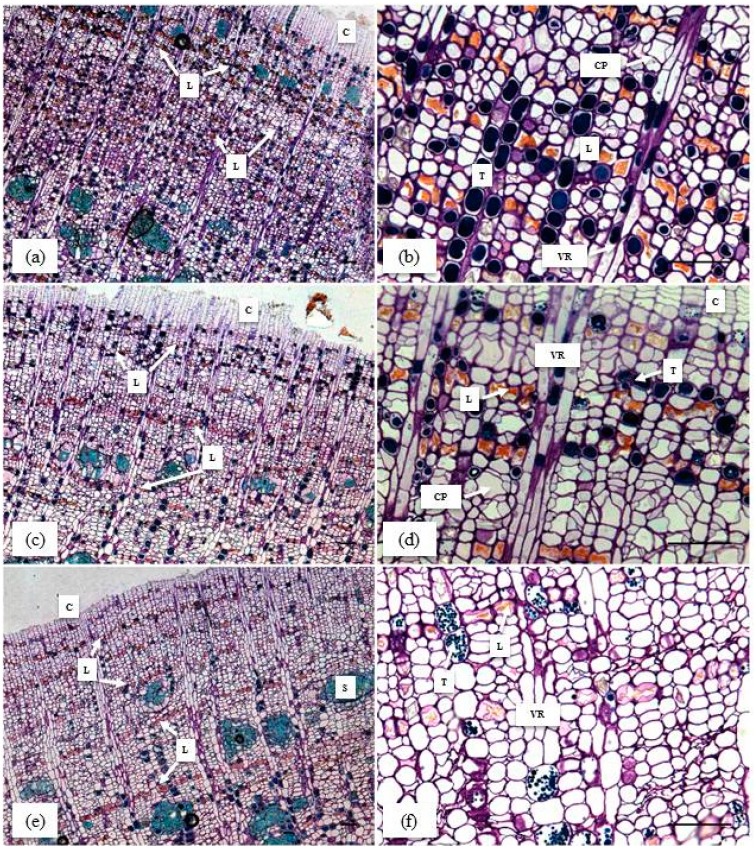
Histo-cytological description of bark tissues from healthy and Tapping Panel Dryness (TPD)-affected trees for high latex metabolism clone PB 260. The histological sections were stained with Oil Red O. Cross-sections of healthy bark (**a**,**b**); reactive oxygen species (ROS)-TPD bark (**c**,**d**); and brown bast (BB)-TPD bark (**e**,**f**); at different magnifications (5× and 20×, respectively) were annotated: C. cambium; L. Laticiferous cells; P. conducting phloem; VR. vascular ray; T. tannin cells; S. sclereid (stone cells).

**Figure 8:**
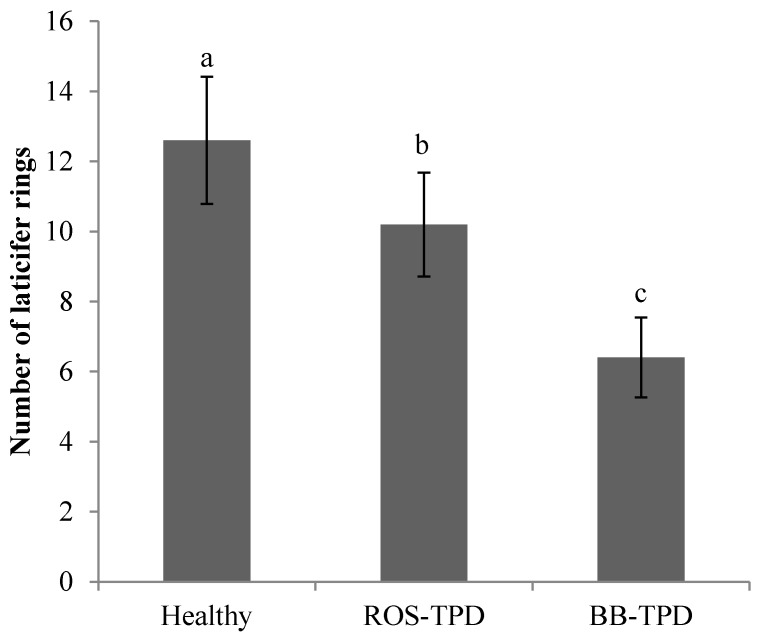
Number of laticifer rings in healthy, Tapping Panel Dryness (TPD)-affected trees, and brown bast (BB)-TPD-affected trees for high latex metabolism clone PB 260. The ANOVA test and the Newman–Keuls test were used in the statistical analyses (*p* < 0.05). Data with different letters (a, b, c) are significantly different at the level of 5%.

A histo-cytological analysis was carried out on healthy (Figure 1a) and two types of TPD-affected trees for clone PB 260. The occurrence of brown bast-TPD was observed after bark scraping (Figure 1c,f,i). The transversal cut of bark was visualised on a section approaching the cambium (Figure 7a,c,e). Oil Red O was used to stain latex in red, which made laticifers easy to spot with low and high magnification of bark samples, at 5× and 20× respectively (Figure 7a–f). Parenchymatous cells possessing tannins coloured by Toluidine blue were mainly arranged around the laticifers (Figure 7b,d,f). Surprisingly, some large sclereids were observed in the vicinity of cambium (Figure 7e). Vascular ray cells were easily distinguished and organized into 2–3 cell layers transversally across the bark from xylem to phloem (Figure 7b,d,f). Several sieve tubes (star-shaped cells) forming conducting phloem were found near laticifers and cambium (Figure 7b,d). Fewer laticifer mantels were visible in BB-TPD, along with deformation of tannin cells and small crushed sieve tubes (Figure 7f). Laticifer mantels were counted revealing a significant decrease in their number in ROS- and BB-TPD trees (Figure 8). Healthy and ROS-TPD trees showed a large number of laticifer mantels, 12.6 ± 1.82 and 10.2 ± 1.48, respectively. By contrast, BB-TPD trees had a small number of laticifer mantels (6.4 ± 1.14) (Table S8).

### 2.7. Fold Change in the Transcript Abundance of Genes Involved in the Ethylene Biosynthesis and Signalling Pathways and ROS-Scavenging System under Tapping Panel Dryness

**Figure 9:**
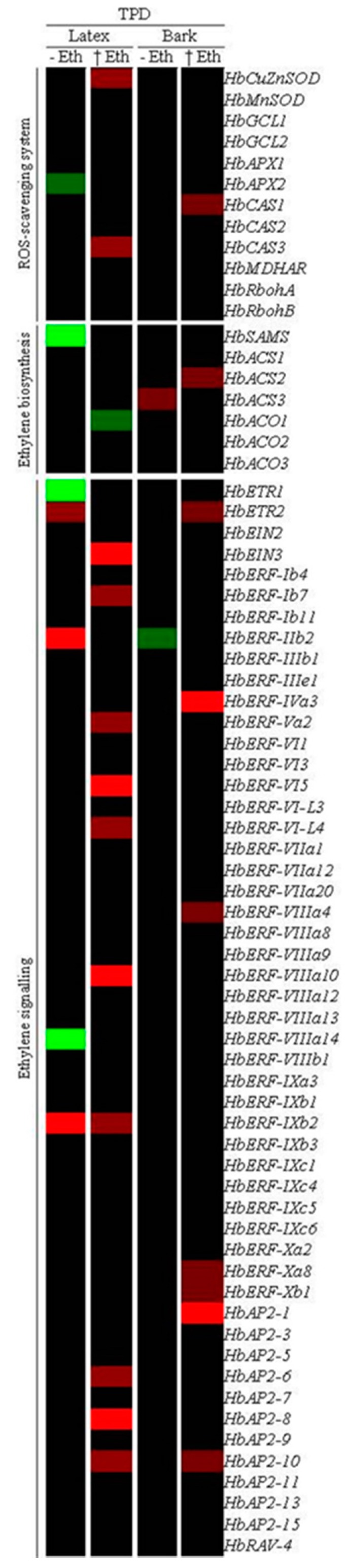
Fold change profile of 70 genes of *Hevea brasiliensis* involved in reactive oxygen species (ROS)-scavenging system, ethylene biosynthesis and signalling during the occurrence of Tapping Panel Dryness (TPD) in 6-year-old mature trees. Values are ratio between TPD and healthy plants without (−Eth) or with ethephon (+Eth) stimulation for six biological replicates. Values of relatives transcript abundance was performed with an ANOVA followed by the Student Newman–Keuls test. Upregulated genes were shown in bright red for a threshold value ≥5; dark red for value <5, and down-regulated genes were shown in bright green for a threshold value ≤0.2; dark green for value >0.2. The non-significant genes are shown in dark colour.

The transcript abundance ratio of 70 *Hevea brasiliensis* genes comparing TPD-affected and healthy trees was analysed in latex and bark of 6-year-old mature trees (Figure 9 and Table S9). These genes were selected for their potential involvement in TPD according to the literature: ethylene biosynthesis (7 genes) and signalling pathways (53 genes including Ethylene Response Factor (ERF)) and ROS-scavenging system (12 genes). Twenty-seven genes were differentially regulated in latex (16 genes), bark (8 genes) and both tissues (3 genes) under TPD for trees cultivated in the absence or presence of ethephon. Three genes were regulated in both latex and bark of TPD-affected trees: *HbETR2*, *HbERF-IIb2* (up-regulated in latex and down-regulated in bark) and *HbAP2-10*. In latex, 3 genes (*HbETR2*, *HbERF-IIb2*, and *HbERF-IXb2*) were induced by TPD as opposed to 4 inhibited genes (*HbAPX2*, *HbSAMS*, *HbERF-VIIIa14*, and *HbETR1*) in non-stimulated trees. By contrast, 12 genes (*HbCuZnSOD*, *HbCAS3*, *HbEIN3*, *HbERF-Ib7*, *HbERF-Va2*, *HbERF-VI5*, *HbERF-VI-L4*, *HbERF-VIIIa10*, *HbERF-IXb2*, *HbAP2-6*, *HbAP2-8*, and *HbAP2-10*) were induced by TPD as opposed to 1 inhibited gene (*HbACO1*) in stimulated trees. In bark, 2 genes (*HbACS3* and *HbERF-Ib2*) were induced and inhibited by TPD, respectively, in non-stimulated trees. Meanwhile, 9 genes (*HbCAS1*, *HbACS2*, *HbETR2*, *HbERF-IVa3*, *HbERF-VIIIa4*, *HbERF-Xa8*, *HbERF-Xb1*, *HbAP2-1*, and *HbAP2-10*) were induced by TPD in stimulated trees.

## 3. Discussion

### 3.1. High Latex Metabolism Clones Are More Susceptible to TPD

The TPD susceptibility of clones is related to their growth and latex metabolism [33]. The identification of this phenotype requires several years of latex harvesting to be determined in plantations under standard harvesting systems [8,18]. Clone PB 260 is a quick-starter clone that can be tapped as early as 4 to 6 years after planting depending on eco-climatic conditions. This clone is characterized by high vigour and a high intrinsic latex metabolism. By contrast, clone SP 217 is a slow-starter that requires ethephon application to stimulate its low latex metabolism. In this study, clones could be distinguished through their TPD susceptibility, monitored as the dry cut length, within two years using a high tapping frequency with ethephon stimulation (d1 ET 12/y). Trees of clones PB 260 and SP 217 were affected by TPD after 5 and 24 months of tapping, respectively, with clone RRIM 600 clearly showing an intermediate phenotype. This early clonal effect was confirmed by the Bonferroni test, whereas tapping and ethephon effects only were significant after three years of tapping. Daily tapping (d1) caused stress in laticifers. The lower latex production per tapping per tree was clearly observed for d1 compared with d2, which is the necessary time for full latex regeneration between two tappings.

Surprisingly, the occurrence of TPD did not result in a decrease in latex production over the duration of this study. Of course, such latex production was not likely to be sustainable, since a drop in dry rubber content began in the third year of production. The continued latex production when partial TPD occurred might be explained by the large latex-producing zone of the tapping panel involved in latex flow and only local oxidative stress in part of the tapping cut. Latex diagnosis is an efficient method for early detection of laticifer dysfunctioning. Typically, an increase in sucrose content and a decrease in thiols content were observed. When TPD symptoms occurred significantly, all the latex diagnosis parameters fell slightly but significantly, revealing a general decrease in metabolic activity in the laticifers.

TPD occurrence increased regularly during the plantation cycle, and the frequency of its occurrence depended dramatically on the latex metabolism of the clones, and environmental and harvesting conditions. As TPD occurrence was spread over time, its quantification remained complicated and hampered any genetic analysis of TPD tolerance. Clones with a high latex metabolism showed earlier TPD occurrence than those with a low latex metabolism when using a harvesting system with a high tapping frequency (d1) and ethephon stimulation (ET 12/y). This suggests that dry cut length measurement could be used as a phenotyping parameter for trees subjected to this stressful harvesting system. Monitoring the dry cut length could be used to distinguish a population derived from a cross between TPD susceptible and tolerant clones. Quantitative Trait Loci (QTL) detection has been successfully carried out to understand the genetic determinism of South American Leaf Blight (SALB) in rubber trees [34,35]. This approach could thus be considered to detect QTLs and underlying genes related to TPD and for selecting new TPD-tolerant clones.

### 3.2. Involvement of the Antioxidant System in the Onset of TPD

This study focused on the TPD form involving oxidative stress generated by harvesting systems. This ROS-TPD type resulted from physiological fatigue of laticifers observed through a drop in sucrose, inorganic phosphorus and antioxidant components such as thiols. Catalase activity was reported to disappear in parallel with a large reduction in SOD activity, and thiol and ascorbate contents [7], which was partially confirmed in this study with the drop in thiol content after TPD occurrence. Abnormal activity of high lutoidic NADPH oxidase is the main source of toxic oxygen production with harmful effects on the lipid membrane in lutoids [7]. During this period, the consumption of oxygen by NADH-Cytochrome-*c*-oxidoreductase from lutoids is particularly high [36]. Thus, hypoxia conditions in laticifers have been suggested in TPD-affected trees. Moreover, control of NADPH oxidase enzymatic activity depends not only on *Rboh* genes but also on ethylene-response genes. NADPH oxidase (RbohD) was needed for the ROS-responsive expression of *Arabidopsis* ERF6 via a calcium-dependent manner [37]. Among the transcription factors studied in *Hevea*, *HbERF-VII* was recently linked to potential hypoxia-responsive genes in laticifers [32]. This ERF might play an important role in the fermentative pathway of the latex metabolism involving alcohol dehydrogenase (ADH). The latter enzyme is also known to be induced by NADPH oxidase [38]. The relative transcript abundance of ROS-scavenging genes was higher in bark than in latex but no differential gene expression was noticed in response to ethephon and TPD occurrence, except the inhibition of *HbAPX2* in latex tissue (Figures S1 and S2). Post-transcriptional regulation of ROS-scavenging systems was assumed, as little transcriptional regulation and dramatic biochemical changes were observed during TPD occurrence.

### 3.3. Involvement of Ethylene Biosynthesis and Signalling in the Onset of TPD

Ethylene is involved in many aspects of latex production, which makes this phytohormone an essential factor of ROS-TPD, which is often induced by over-application of ethephon on rubber trees. Endogenous production of ethylene has been reported during latex harvesting by tapping and ethephon stimulation [6,39], and also during the occurrence of brown bast-TPD [12]. Ethylene influences changes at the morphological, cellular, and molecular levels [6]. For instance, ethylene causes bark thickening that leads to BB-TPD. This study confirmed that ROS-TPD and BB-TPD trees display a small number of concentric mantels of laticifers; this anomaly is related to the multiplication of parenchyma cells at aggravated levels of TPD [12]. This drop in the number of laticifers in TPD-affected suggested some modification of the cambial activity compared to healthy trees. Recently, Thai researchers reported that the double actions of abscisic acid (ABA) and auxin led to down-regulation of Auxin Response Factor (ARF) 6 and ARF8, and consequently to the attenuation of vascular development in ethylene-stimulated trees [40]. They concluded that the cumulative effect of Homeodomain Leucine Zipper (HD-ZIP) III, ARF6, and ARF8 down-regulation by long-term ethylene treatment may lead to TPD syndrome.

The transcript abundance analysis for genes involved in ethylene biosynthesis showed a differential expression in latex and in bark tissues. Globally, the relative transcript abundance of these genes was higher in bark than in latex (Figures S1 and S2). In latex, down-regulation of the *HbSAMS* gene suggested a predominant control of this gene during TPD. Concurrently, the *HbACS3* gene had a high transcript abundance in both tissues and it was dramatically induced by TPD in bark. This result suggests that ethylene could be potentially synthesized in bark but less so in latex. In addition, we did not observe any changes in expression of *HbACO* genes in latex, which encode the last enzyme to convert ACC into ethylene. Post-transcriptional regulation of the genes involved in the ethylene biosynthesis pathway was likely to be the prominent hypothesis. Control of ACS and ACO protein stability is widely described. The ACS5 protein was negatively regulated by ETO1 [41,42] via ubiquitin/26S proteasome degradation [43]. For ACOs, the activity of RUB (related to ubiquitin) and RCE (RUB-conjugating enzyme) was involved in the activation of ubiquitin ligase to promote proteasome-mediated degradation of ACOs [44,45].

Ethylene was differentially perceived and transduced in latex and in bark during ROS-TPD. In general, the relative transcript abundance of *HbETR1*, *HbETR2*, *HbEIN2* and *HbEIN3* was higher in bark than in latex (Figures S1 and S2). The *HbETR2* gene had a high transcript abundance in both tissues. On the other hand, the *HbEIN3* gene had a higher transcript abundance in latex than in bark. These results suggest that ethylene biosynthesis mainly occurs in bark and could be perceived in both tissues. When TPD occurred, *HbETR1* and *HbETR2* showed an opposed response in laticifers, with strong inhibition and induction, respectively. It is known that ETR1 and ETR2 are differentially regulated by ethylene [46]. This dissimilarity of ethylene perception in latex and bark could result in different responses of downstream genes, as shown in this study. Indeed, genes involved in ethylene signalling were not similarly regulated in both tissues during TPD. In general, the transcript abundance of *HbERF-I* was higher in bark than in latex, while *HbERF-VIII* had a high transcript abundance in both tissues (Figures S1 and S2). ERF-VIII is involved in programmed cell death (PCD) [47,48]. Accumulation of *HbERF-VIII* transcripts suggests a potential involvement of early PCD in ROS-TPD. At transcript level, TPD seems to have a repression effect for some genes. The transcript abundance of *HbERF-IXc4* in latex and two genes of group VIII (*HbERF-VIIIa4* and *HbERFVIIIa10*) in bark was significantly induced by ethephon but not in the case of TPD. Besides this transcriptional regulation, post-transcriptional regulation is expected for several ERF groups. For example, the ubiquitination of protein degradation has been demonstrated for ERF-VII [49]. Alternative splicing has also been reported to control isoforms of DREB2A genes in several plant species, which belong to the ERF-III group in our study [50,51,52].

### 3.4. Identification of Expression Marker Genes during ROS-TPD

Of the 70 genes related to ROS-scavenging systems, and ethylene biosynthesis and signaling pathways, this study highlighted 27 TPD-expression marker genes in latex and bark. The expression marker genes differed between latex and bark except for two genes. This suggests tissue-specific regulation of genes involved in ROS-TPD occurrence. Regardless of the tissue, more expression marker genes of ROS-TPD were identified in ethephon-stimulated trees (22) than non-stimulated trees (9), revealing a complex regulation of ROS-TPD. Among the expression marker genes, several had a putative important function predicted by phylogenetic analysis, such as *HbERF-IIb2*, *HbERF-VIIIa14* and *HbERF-IXb2* [32]. *HbERF-IIb2* is orthologous to OCTADECANOID RESPONSIVE ARABIDOPSIS AP2/ERF (ORA) 47, which controls jasmonate biosynthesis. Several expression marker genes were also related to the programmed cell death (PCD). ERF group VIII is renowned for Programmed Cell Death (PCD) induction during ectopic over-expression [48]. The repression of *HbERF-VIIIa14*, a repressor-type ERF, potentially displayed PCD regulation. Recently, ERF-IXb was identified as an ERF subgroup involved in PCD [53,54]. The gene *HbERF-IXb2* was not regulated by ethylene [32]. Transcripts of 4 of the 11 *HbAP2* genes were also significantly accumulated including *HbAP2-6*, an ortholog to WRI1 [32], and *HbAP2-10*, which was an expression marker gene in both the bark and latex. This regulation of *HbAP2* genes during TPD suggested metabolic and developmental regulation. All these expression marker genes represent good candidate genes for studying the allelic variability and development of genetic markers associated with susceptibility or tolerance of TPD.

## 4. Experimental Section

### 4.1. Plant Material

Latex and bark tissues were collected for three years after the first tapping from 6-year-old mature rubber trees of three *Hevea* clones (PB 260, RRIM 600, SP 217) at the Sembawa Research Centre of the Indonesian Rubber Research Institute. The latex harvesting system was a half spiral tapping (S/2), with a tapping frequency of 1 (d1), 2 (d2) and 4 days (d4), respectively. Ethephon (Eth) was applied at 2.5% to the tapping panel of the bark at various frequencies (0 (0/y), 12 (12/y) and 24 (24/y) times a year). Three replicates of one tree each were observed for each combination (clone × tapping frequency × ethephon treatment). The length of dry cut was observed just after tapping before tissue sampling in order to measure visually the tapping panel dryness gradient. Samples of healthy and TPD-affected trees were then collected 24 h after ethephon treatment. Sample tissues were collected and immediately frozen in liquid nitrogen and stored at −80 °C until RNA extraction.

### 4.2. Histo-Cytological Analysis

Histological samples of TPD-affected trees were collected 2 cm below the tapping cut using a punch without damaging the cambium. The samples were fixed as described by [55]. After inclusion in resin, the cross-sections obtained with a microtome were stained with Toluidine Blue and the Oil Red O staining method adapted from Lillie and Ashburn [56] by the PHIV platform (CIRAD, Montpellier, France). The image bar scale was defined using image analysis software (ImageJ, Bethesda, MD, USA). The number of concentric mantels of laticifers was manually calculated. Statistical analysis was performed using an ANOVA followed by a Newman–Keuls test.

### 4.3. Measurement of the Dry Cut Length and Latex Diagnosis

The gradient of tapping panel dryness was assessed by measuring the dry cut length (DCL). The DCL was measured by visual estimation of the percentage of half-spiral tapping cut without latex flow instantly after tapping. Latex diagnosis parameters *i.e.*, sucrose (Suc), inorganic phosphorous (Pi), and thiol contents (R-SH) were measured from March to May after the rainy season and before leaf senescence in order to avoid any physiological variation. Fresh latex (0.5 mL) was supplemented with 2.5% trichloroacetic acid (TCA) with 4.5 mL of volume to induce protein precipitation, and then with 0.2% phosphotungsic acid (PTA) to coagulate rubber particles. This clear latex serum was then used for the measurement of sucrose, inorganic phosphorous and thiols using the Dische anthrone method [57], the Taussky dan Shorr molybdate ammonium method [58], and the McMullen acid dithiobisnitrobenzoat (DTNB) method [59], respectively.

### 4.4. Total RNA Isolation

Total RNAs were isolated using the caesium chloride cushion method adapted from Sambrook [60] by Duan and Coll. [61]. One gram of fresh matter was ground and transferred to a tube containing 30 mL of extraction buffer consisting of 4 M guanidium isothiocyanate, 1% sarcosine, 1% polyvinylpyrrolidone and 1% β-mercapto-ethanol. After homogenization, tubes were kept on ice and then centrifuged at 10,000× *g* at 4 °C for 30 min. The supernatant was transferred to a new tube containing 8 mL of 5.7 M CsCl. Ultracentrifugation in a swinging bucket was carried out at 89,705× *g* at 20 °C for 20 h. The supernatant and caesium cushion were discarded whilst the RNA pellet was washed with 70% ethanol. After 30 min of air drying, the pellet was dissolved in 200 µL of sterile water. Although DNA could not cross the caesium cushion for this centrifugation condition, a DNAse treatment was performed using TurboDNAse (Ambion, Life Technologies, Carlsbad, CA, USA). DNA contamination was then checked by PCR amplification using primers of the Actin gene including the intron sequence. RNAs were conserved at −80 °C.

### 4.5. Primer Design and Analysis of Transcript Abundance by Real-Time RT-PCR

Real-time RT-PCR experiments followed recommendations specified by Udvardi *et al.* [62]. Specific primers have already been designed for ethylene biosynthesis and signalling pathways, and ROS-scavenging systems [61,62,63,64]. Several rules were applied in order to reduce the risk of error in relative gene expression data. The integrity of total RNA was checked by electrophoresis. Primers were designed at the 3′ side of each sequence in order to reduce the risk of error due to short cDNA synthesis using the Primer 3 module of Geneious (Biomatters Ltd., Auckland, New Zealand). Real-time PCR amplification and the fusion curve were carried out using a mix of cDNAs in order to check the specificity of each pair of primers. Sequencing of the PCR amplicon was carried out to verify the product sequence. Primer sequences are listed in Table S10.

cDNAs were synthesized from 2 μg of total RNA to the final 20 μL reaction mixture using a RevertAidTM M-MuLV Reverse Transcriptase (RT) kit according to the manufacturer’s instructions (MBI, Fermentas, Burlington, ON, Canada). Full-length cDNA synthesis was checked on each cDNA sample by PCR amplification of the Actin cDNA using primers at the cDNA ends. Quantitative gene expression analysis was finally carried out by real-time RT-PCR using a Light Cycler 480 (Roche, Basel, Switzerland). Real-time PCR reaction mixtures consisted of 2 µL RT product cDNA, 0.6 µL of 5 µM of each primer, and 3 µL 2× SYBR green PCR master mix (LightCycler^®^ 480 SYBR Green I Master, Roche Applied Sciences, Basel, Switzerland) in a 6-µL volume. PCR cycling conditions comprised one denaturation cycle at 95 °C for 5 min, followed by 45 amplification cycles (95 °C for 20 s, 60 °C for 15 s, and 72 °C for 20 s). Expression analysis was performed in a 384-well plate. Samples were loaded using an automation workstation (Biomek NX, Beckman Coulter, Brea, CA, USA).

Real-time PCR was carried out for eleven housekeeping genes in order to select the most stable gene as the internal control for all types of sample used (*HbelF1Aa*, *HbUBC4*, *HbUBC2b*, *HbYLS8*, *HbRH2b*, *HbRH8*, *HbUBC2a*, *HbalphaTub*, *Hb40S*, *HbUbi*, *HbActin*) (Table S11). *HbRH2b* was selected as the best reference gene according to its stability in tissues from various treatments in mature trees and juvenile trees. The homogeneity of the *HbRH2b* gene Cp (crossing point-PCR-cycle) confirmed that it could be used as an internal reference gene. The *HbRH2b* gene was amplified in each reaction plate in parallel with target genes. The transcript abundance level for each gene was relatively quantified by normalization with the transcript abundance of the reference *HbRH2b* gene. Relative transcript abundance took into account primer efficiencies. All the normalized ratios corresponding to transcript accumulation were calculated automatically by Light Cycler Software version 1.5.0 (Manufacturer Roche Diagnostics Ltd., Rotkreuz, Switzerland) provided by the manufacturer using the following calculation: Normalized Ratio = Efficiency ^−Δ*(Cp target − Cp RH2b)*^.

### 4.6. Statistical Analysis for the Comparison of Relative Transcript Abundance and for the Analysis of Interactions between the Effect of Ethephon, TPD and a Combination of both in Mature Trees

Dry cut length values and latex diagnosis physiological data (sucrose, inorganic phosphorus, and thiols) were the mean of three biological replicates. The statistical analysis was carried out on raw data using an ANOVA followed by a Bonferroni test for dry cut length and by Fisher’s *F*-test for latex diagnosis data. The values were considered as significantly different when *p* value ≤0.05. The level of interaction between clone, tapping, ethephon, the combination of two, *i.e.*, clone × tapping, clone × ethephon, tapping × ethephon, and a combination of the three (clone × tapping × ethephon) was assessed for the DCL. The level of interaction between clones (PB 260 × SP 217, PB 260 × RRIM 600, and RRIM 600 × SP 217), tapping frequencies (d1 × d2, d1 × d4, and d2 × d4), and ethephon stimulation frequencies per year (12/y × 0/y, 12/y × 24/y, 24/y × 0/y) was equally assessed.

Each relative transcript abundance value was the mean of six biological replicates. Statistical analysis was performed after logarithmic transformation of raw data. The analysis of relative transcript abundances of TPD-affected and healthy trees was carried out using an ANOVA followed by a Newman–Keuls test. The level of interaction between ethephon (Eth), tapping panel dryness (TPD) and a combination of both (Eth × TPD) was assessed for each tested gene. The analysis generated variance tables that included *F* values for each interaction and the corresponding *p* values were noted as follows: <0.001 (***); <0.01 (**); <0.05 (*); <0.1 (°).

## 5. Conclusions

Environmental constraints are known to induce the production of ROS and ethylene. Monitoring TPD occurrence is a simple method to highlight ROS-related latex cell dysfunctions. The dramatic regulation of genes involved in ethylene perception and signalling during TPD reinforces the critical role of this hormone in plant defence. The putative functions of some *HbAP2/ERFs* also suggested the involvement of other hormones and developmental processes such as jasmonate, and PCD and cambial activity, respectively. Beyond improving our knowledge of the TPD, this study contributes to a better understanding of the role of transcription factors and in particular ethylene signalling in perennial species. Unveiling the role of AP2/ERF transcription factors in the plant defence processes in *Hevea*, may help molecular breeders in improving tolerance to abiotic and harvesting stress.

## Supplementary Materials

Supplementary materials can be found at http://www.mdpi.com/1422-0067/16/08/17885/s1.
