# Supplementary material for: Involvement of Ethylene in the Latex Metabolism and Tapping Panel Dryness of Hevea brasiliensis
Source: Int J Mol Sci. 2015 Aug 4;16(8):17885–908. doi: 10.3390/ijms160817885 (PMC4581227; doi:10.3390/ijms160817885)
Supplement: Supplementary file 1 [file ijms-16-17885-s001.zip › ijms-84942-Supplementary Information/ijms-84942-Supplementary figures S1 and S2 1.pdf]

Supplementary Information

	Gene	- Eth		+Eth	
		Healthy	TPD	Healthy	TPD
ROS-scavenging system	*HbCuZnSOD*	ND	ND	2,42E-03^{b}	7,33E-03^{a}
	HbMnSOD	6,35E-02^{a}	ND	6,42E-03^{a}	1,18E-02^{a}
	HbGCL1	1,01E-08^{a}	1,05E-08^{a}	1,02E-08^{a}	1,02E-08^{a}
	HbGCL2	1,44E-01^{a}	1,10E-01^{a}	1,15E-01^{a}	1,24E-01^{a}
	HbAPX1	1,01E-08^{a}	1,05E-08^{a}	1,02E-08^{a}	1,02E-08^{a}
	HbAPX2	6,54E-02^{a}	4,03E-02^{a}	ND	3,04E-03^{b}
	HbCAS1	ND	2,48E-03^{a}	ND	ND
	HbCAS2	1,01E-08^{a}	1,05E-08^{a}	1,02E-08^{a}	1,02E-08^{a}
	HbCAS3	1,04E-01^{a}	ND	5,05E-03^{b}	1,06E-02^{b}
	HbMDHAR	1,55E-01^{a}	1,94E-02^{a}	2,25E-02^{a}	4,17E-02^{a}
	HbRbohA	1,05E-03^{a}	4,56E-03^{a}	4,97E-04^{a}	5,72E-04^{a}
	HbRbohB	6,30E-01^{a}	1,24E+00^{a}	2,71E-01^{a}	7,93E-01^{a}
Ethylene biosynthesis	*HbSAMS*	8,41E-05^{a}	6,41E-06^{b}	1,19E-05^{b}	1,19E-05^{b}
	HbACS1	1,82E-03^{a}	5,45E-03^{a}	4,81E-03^{a}	1,33E-03^{a}
	HbACS2	ND	ND	ND	6,06E-05^{a}
	HbACS3	1,42E-06^{b}	ND	3,01E-04^{a}	3,01E-04^{a}
	HbACO1	5,19E-04^{a}	5,77E-04^{a}	1,69E-04^{b}	1,44E-04^{c}
	HbACO2	5,36E-03^{a}	1,30E-02^{a}	1,79E-03^{a}	6,48E-03^{a}
	HbACO3	6,33E-05^{a}	ND	ND	6,74E-06^{b}
Ethylene signalling	*HbETR1*	3,52E-03^{a}	6,58E-04^{b}	1,21E-03^{b}	2,86E-03^{ab}
	HbETR2	8,07E+00^{b}	1,46E+01^{a}	4,17E+00^{c}	4,44E+00^{c}
	HbEIN2	5,32E+00^{a}	6,55E+00^{a}	6,45E+00^{a}	6,94E+00^{a}
	HbEIN3	1,17E-02^{b}	1,42E-02^{ab}	1,20E-02^{b}	1,62E-01^{a}
	HbERF-Ib4	4,83E-03^{b}	ND	8,07E-03^{ab}	3,08E-02^{a}
	HbERF-Ib7	7,09E-03^{b}	5,78E-03^{b}	1,34E-02^{b}	5,38E-02^{a}
	HbERF-Ib11	6,02E-05^{a}	ND	3,38E-04^{a}	6,81E-04^{a}
	HbERF-IIb2	7,14E-03^{b}	6,51E-02^{a}	2,34E-02^{a}	2,94E-02^{a}
	HbERF-IIIb1	4,12E-04^{a}	2,90E-04^{a}	1,61E-04^{a}	1,25E-04^{a}
	HbERF-IIIe1	1,30E-04^{a}	6,75E-06^{a}	1,07E-04^{a}	3,58E-04^{a}
	HbERF-IVa3	1,01E-02^{a}	5,94E-03^{a}	1,10E-02^{a}	2,20E-02^{a}
	HbERF-Va2	9,06E-03^{a}	6,07E-02^{a}	3,79E-03^{a}	1,72E-02^{a}
	HbERF-VI1	4,27E-03^{a}	3,50E-03^{a}	4,41E-03^{a}	1,24E-02^{a}
	HbERF-VI3	3,07E-03^{a}	1,26E-03^{a}	1,10E-03^{a}	5,27E-03^{a}
	HbERF-VI5	9,73E-04^{b}	ND	1,82E-03^{b}	2,51E-02^{a}
	HbERF-VI-L3	1,35E-02^{a}	1,12E-02^{a}	4,05E-03^{a}	1,72E-02^{a}
	HbERF-VI-L4	7,39E-02^{ab}	6,91E-02^{ab}	3,93E-02^{b}	9,08E-02^{a}
	HbERF-VIIa1	ND	2,15E-03^{a}	1,08E-03^{a}	1,32E-03^{a}
	HbERF-VIIa12	3,19E-03^{a}	1,89E-03^{a}	1,97E-03^{a}	3,86E-04^{a}
	HbERF-VIIa20	1,35E-01^{a}	1,85E-01^{a}	8,48E-02^{a}	1,31E-01^{a}
	HbERF-VIIIa4	4,09E+00^{a}	5,12E+00^{a}	2,51E+00^{a}	3,07E+00^{a}
	HbERF-VIIIa8	5,69E-01^{a}	1,56E+00^{a}	2,48E-01^{a}	3,17E-01^{a}
	HbERF-VIIIa9	6,99E-01^{ab}	1,17E+00^{a}	2,47E-01^{c}	4,27E-01^{bc}
	HbERF-VIIIa10	1,23E-02^{ab}	1,93E-02^{ab}	9,33E-03^{b}	5,16E-02^{a}
	HbERF-VIIIa12	2,57E-03^{a}	1,26E-03^{a}	1,06E-03^{a}	2,30E-02^{a}
	HbERF-VIIIa13	7,89E-03^{a}	2,05E-02^{a}	1,95E-02^{a}	1,46E-02^{a}
	HbERF-VIIIa14	7,23E-03^{b}	2,16E-04^{c}	1,29E-02^{ab}	7,27E-02^{a}
	HbERF-VIIIb1	8,84E-03^{a}	1,18E-02^{a}	2,20E-03^{a}	2,21E-03^{a}
	HbERF-IXa3	5,66E-03^{a}	7,32E-03^{a}	6,29E-03^{a}	6,77E-03^{a}
	HbERF-IXb1	5,82E+00^{a}	7,88E-02^{a}	9,66E-03^{a}	7,32E-03^{a}
	HbERF-IXb2	8,44E-03^{b}	5,49E-02^{a}	2,11E-03^{c}	4,65E-03^{bc}
	HbERF-IXb3	2,45E-04^{a}	1,90E-04^{a}	7,71E-05^{a}	1,94E-04^{a}
	HbERF-IXc1	4,46E-03^{a}	3,80E-03^{a}	2,64E-03^{a}	2,68E-03^{a}
	HbERF-IXc4	1,02E-01^{b}	7,44E-02^{b}	4,72E-01^{a}	3,03E-01^{ab}
	HbERF-IXc5	8,42E-05^{a}	2,11E-05^{a}	1,50E-04^{a}	1,65E-04^{a}
	HbERF-IXc6	6,84E-04^{a}	7,78E-04^{a}	2,78E-04^{a}	5,55E-04^{a}
	HbERF-Xa2	1,99E-02^{a}	5,75E-02^{a}	6,05E-02^{a}	3,45E-02^{a}
	HbERF-Xa8	4,10E-03^{a}	ND	4,78E-03^{a}	5,73E-03^{a}
	HbERF-Xb1	6,30E-03^{b}	1,54E-02^{ab}	8,07E-03^{a}	1,78E-02^{a}
	HbAP2-1	3,77E-04^{a}	1,44E-04^{a}	1,38E-04^{a}	7,16E-05^{a}
	HbAP2-3	2,50E-02^{a}	5,10E-02^{a}	2,49E-02^{a}	7,79E-03^{a}
	HbAP2-5	1,62E-03^{a}	9,42E-04^{a}	8,53E-04^{a}	7,87E-04^{a}
	HbAP2-6	1,71E+00^{a}	1,69E+00^{a}	7,43E-02^{c}	2,48E-01^{b}
	HbAP2-7	5,97E-03^{a}	7,79E-03^{a}	2,92E-03^{a}	5,62E-03^{a}
	HbAP2-8	2,24E-03^{a}	1,46E-03^{a}	2,85E-04^{a}	2,54E-03^{a}
	HbAP2-9	3,02E-02^{a}	4,31E-02^{a}	1,69E-03^{a}	7,39E-03^{a}
	HbAP2-10	2,63E-02^{a}	1,17E-02^{b}	1,14E-02^{b}	3,41E-02^{a}
	HbAP2-11	5,46E-03^{a}	9,34E-03^{a}	1,08E-03^{a}	4,38E-03^{a}
	HbAP2-13	2,17E-03^{a}	4,51E-03^{a}	4,30E-04^{a}	3,92E-04^{a}
	HbAP2-15	8,80E-03^{a}	5,43E-03^{a}	2,51E-03^{a}	1,91E-03^{a}
	HbRAV-4	3,52E-04^{a}	7,45E-04^{a}	1,43E-04^{a}	1,23E-04^{a}

Figure S1. Relative transcript abundance profile of 70 genes of *Hevea brasiliensis* involved in ROS-scavenging system, ethylene biosynthesis and signalling during the occurrence of Tapping Panel Dryness in latex of 6-year-old mature trees. The relative transcript abundance was measured by real-time RT-PCR. Tapping without ethephon (−Eth) and with ethephon (+Eth) were applied in both studied tissues. Values are the means of three biological replicates. Heat map representation was used for values ranging as follows ≥ 1, 10^{-1}, 10^{-2}, 10^{-3} and $\leq 10^{-4}$ from dark to light green. Values of relative transcript abundances were analysed with XLSTAT after LOG(X) transformation. The statistical analysis was performed with an ANOVA followed by the Student Newman–Keuls test.

	Gene	-Eth		+Eth	
		Healthy	TPD	Healthy	TPD
ROS-scavenging system	*HbCuZnSOD*	3,48E-04^{a}	8,15E-05^{ab}	2,11E-05^{b}	3,17E-05^{b}
	HbMnSOD	2,27E+00^{a}	3,12E+00^{a}	1,11E+00^{b}	1,41E+00^{b}
	HbGCL1	1,83E-02^{b}	2,25E-02^{ab}	5,54E-02^{a}	7,36E-02^{ab}
	HbGCL2	1,03E-01^{a}	1,48E-01^{a}	1,21E-01^{a}	1,08E-01^{a}
	HbAPX1	5,32E+00^{a}	4,94E+00^{ab}	3,43E+00^{b}	3,98E+00^{b}
	HbAPX2	1,16E-02^{a}	1,33E-02^{a}	7,17E-03^{a}	9,35E-03^{a}
	HbCAS1	2,79E-03^{c}	2,50E-03^{bc}	8,43E-03^{a}	1,96E-02^{b}
	HbCAS2	6,86E-03^{c}	7,48E-03^{c}	4,46E-02^{a}	1,05E-01^{b}
	HbCAS3	2,49E-01^{c}	2,34E-01^{bc}	1,43E+00^{a}	2,59E+00^{ab}
	HbMDHAR	1,70E-02^{c}	1,81E-02^{bc}	2,92E-02^{ab}	2,43E-02^{a}
	HbRbohA	3,45E-01^{a}	2,56E-01^{ab}	1,85E-01^{b}	1,86E-01^{b}
	HbRbohB	2,05E-01^{a}	2,11E-01^{a}	1,56E-01^{a}	1,97E-01^{a}
Ethylene biosynthesis	*HbSAMS*	1,84E-01^{a}	8,98E-02^{a}	3,86E-01^{a}	1,58E-01^{a}
	HbACS1	2,25E-02^{a}	3,43E-02^{a}	1,71E-02^{a}	4,76E-03^{a}
	HbACS2	6,93E-03^{a}	9,11E-03^{a}	3,37E-03^{a}	1,38E-02^{a}
	HbACS3	2,49E-03^{b}	7,11E-03^{a}	3,05E-03^{b}	2,74E-03^{b}
	HbACO1	6,16E-04^{a}	3,23E-03^{a}	2,20E-04^{a}	1,26E-04^{a}
	HbACO2	3,39E-01^{b}	3,77E-01^{b}	1,30E+00^{a}	2,53E+00^{ab}
	HbACO3	3,45E-03^{a}	1,29E-02^{a}	1,67E-03^{a}	2,47E-03^{a}
Ethylene signalling	*HbETR1*	9,23E-02^{a}	7,34E-02^{a}	4,83E-02^{a}	5,69E-02^{a}
	HbETR2	1,07E+00^{a}	1,41E+00^{a}	4,64E-01^{b}	6,34E-01^{c}
	HbEIN2	4,46E-01^{a}	4,57E-01^{a}	2,65E-01^{a}	3,41E-01^{a}
	HbEIN3	1,97E+00^{a}	2,04E+00^{a}	1,42E+00^{a}	1,21E+00^{a}
	HbERF-Ib4	2,42E+00^{a}	3,87E-01^{a}	9,97E-02^{a}	1,95E-01^{a}
	HbERF-Ib7	9,16E+02^{a}	2,19E-01^{a}	3,67E-01^{a}	4,19E-01^{a}
	HbERF-Ib11	6,50E-04^{a}	9,11E-05^{a}	1,82E-04^{a}	2,30E-04^{a}
	HbERF-IIb2	2,01E-04^{c}	8,82E-05^{c}	5,57E-04^{a}	1,37E-03^{a}
	HbERF-IIIb1	1,94E+00^{a}	9,82E-04^{a}	1,05E-04^{a}	1,25E-03^{a}
	HbERF-IIIe1	1,90E-02^{a}	8,41E-03^{a}	1,60E-03^{b}	1,98E-03^{b}
	HbERF-IVa3	1,57E-02^{a}	1,10E-02^{a}	1,77E-03^{a}	1,38E-02^{a}
	HbERF-Va2	3,60E-02^{a}	2,89E-02^{a}	3,62E-03^{b}	2,13E-03^{b}
	HbERF-VI1	6,05E-04^{a}	6,38E-04^{a}	3,72E-05^{a}	2,91E+01^{a}
	HbERF-VI3	4,70E-03^{a}	2,86E-03^{a}	8,02E-03^{a}	5,50E+00^{a}
	HbERF-VI5	4,13E-02^{a}	6,00E-02^{a}	1,52E-02^{a}	3,26E-02^{a}
	HbERF-VI-L3	4,25E-02^{a}	5,47E-02^{a}	5,09E-02^{a}	1,56E-01^{a}
	HbERF-VI-L4	8,33E-03^{a}	1,79E-02^{a}	3,92E-03^{a}	1,40E-02^{a}
	HbERF-VIIa1	3,14E-02^{a}	1,18E-01^{a}	2,78E-03^{b}	4,41E-03^{b}
	HbERF-VIIa12	1,76E-02^{a}	2,89E-02^{a}	1,68E-03^{ab}	8,15E-03^{b}
	HbERF-VIIa20	5,16E-02^{a}	3,42E-01^{a}	2,53E-02^{a}	1,35E+00^{a}
	HbERF-VIIIa4	1,59E-01^{b}	1,55E-01^{b}	1,60E-01^{a}	4,31E-01^{b}
	HbERF-VIIIa8	4,16E-01^{a}	4,32E-01^{a}	1,29E-01^{b}	1,06E-01^{b}
	HbERF-VIIIa9	6,34E-02^{a}	7,54E-02^{a}	5,83E-02^{a}	3,86E-02^{a}
	HbERF-VIIIa10	1,60E-01^{b}	1,30E-01^{b}	3,16E-01^{a}	1,20E+00^{ab}
	HbERF-VIIIa12	6,04E-01^{a}	7,92E-01^{a}	3,56E-01^{a}	2,20E-01^{a}
	HbERF-VIIIa13	1,16E-01^{ab}	1,81E-01^{a}	6,96E-02^{b}	6,91E-02^{b}
	HbERF-VIIIa14	6,91E-02^{a}	8,29E-02^{a}	4,83E-02^{a}	6,23E-02^{a}
	HbERF-VIIIb1	1,59E-04^{a}	3,41E-04^{a}	1,56E+00^{a}	8,34E-05^{a}
	HbERF-IXa3	4,70E-02^{a}	7,97E-02^{a}	5,39E-02^{a}	9,24E-02^{a}
	HbERF-IXb1	4,75E-02^{a}	3,08E-01^{a}	3,56E-02^{a}	4,45E-02^{a}
	HbERF-IXb2	4,50E-03^{ab}	8,88E-02^{a}	1,69E-03^{ab}	4,21E-03^{b}
	HbERF-IXb3	2,04E-03^{a}	1,81E-03^{a}	2,90E-03^{a}	9,29E-03^{a}
	HbERF-IXc1	1,53E-01^{a}	2,48E-01^{a}	8,10E-02^{a}	1,71E-01^{a}
	HbERF-IXc4	2,80E-02^{a}	9,80E-02^{a}	4,50E-02^{a}	2,13E-01^{a}
	HbERF-IXc5	7,34E-03^{b}	1,19E-02^{ab}	3,05E-02^{a}	6,65E-02^{ab}
	HbERF-IXc6	1,77E-02^{a}	4,36E-02^{a}	6,78E-02^{a}	6,51E-02^{a}
	HbERF-Xa2	4,72E-02^{a}	3,69E-02^{a}	9,49E-02^{a}	1,27E-01^{a}
	HbERF-Xa8	5,11E-04^{b}	3,21E-04^{b}	9,19E-04^{a}	3,12E-03^{b}
	HbERF-Xb1	3,89E-04^{a}	2,98E-04^{a}	3,47E-04^{a}	7,66E-04^{a}
	HbAP2-1	7,16E-05^{a}	3,79E-05^{ab}	1,02E-08^{b}	3,21E-05^{c}
	HbAP2-3	7,22E-04^{a}	4,81E-04^{a}	8,89E-04^{a}	1,41E-03^{a}
	HbAP2-5	4,37E-03^{a}	1,48E-03^{a}	2,09E-03^{a}	1,89E-03^{a}
	HbAP2-6	1,26E-02^{a}	6,33E-03^{a}	1,05E-02^{a}	1,86E-02^{a}
	HbAP2-7	3,35E-03^{a}	2,08E-03^{a}	1,68E-04^{b}	1,94E-04^{b}
	HbAP2-8	1,14E-02^{a}	5,92E-03^{ab}	1,16E-03^{ab}	3,38E-03^{b}
	HbAP2-9	7,95E-02^{a}	1,51E-01^{a}	4,07E-02^{a}	4,57E-02^{a}
	HbAP2-10	1,38E-02^{a}	4,63E-03^{a}	1,76E-03^{a}	6,40E-03^{b}
	HbAP2-11	5,23E-04^{a}	3,89E-04^{a}	1,39E-04^{a}	3,33E-04^{a}
	HbAP2-13	1,88E-04^{a}	1,57E-04^{b}	2,25E-05^{ab}	1,07E-04^{b}
	HbAP2-15	7,85E-03^{a}	6,07E-03^{a}	8,14E-04^{b}	5,77E-04^{b}
	HbRAV-4	5,77E-05^{a}	1,47E-04^{a}	3,00E-05^{a}	3,08E-05^{a}

Figure S2. Relative transcript abundance profile of 70 genes of *Hevea brasiliensis* involved in ROS-scavenging system, ethylene biosynthesis and signalling during the occurrence of Tapping Panel Dryness in bark of 6-year-old mature trees. The relative transcript abundance was measured by real-time RT-PCR. Tapping without ethephon (−Eth) and with ethephon (+Eth) were applied in both studied tissues. Values are the means of three biological replicates. Heat map representation was used for values ranging as follows ≥1, 10^{-1}, 10^{-2}, 10^{-3} and ≤10^{-4} from dark to light green. Values of relative transcript abundances were analysed with XLSTAT after LOG(X) transformation. The statistical analysis was performed with an ANOVA followed by the Student Newman–Keuls test.
